# The effects of name and religious priming on ratings of a well-known political figure, President Barack Obama

**DOI:** 10.1371/journal.pone.0180676

**Published:** 2017-06-30

**Authors:** Gary A. Williams, AnaMarie C. Guichard, JungHa An

**Affiliations:** 1 Department of Psychology and Child Development, California State University, Stanislaus, Turlock, California, United States of America; 2 Department of Mathematics, California State University, Stanislaus, Turlock, California, United States of America; University of Akron, UNITED STATES

## Abstract

Priming with race-typed names and religious concepts have been shown to activate stereotypes and increase prejudice towards out-groups. We examined the effects of name and religious word priming on views of a specific and well-known person, President Barack Obama. We predicted that politically conservative participants primed with President Obama’s middle name (Hussein) would rate him more negatively and be more likely to view him as a Muslim than those not shown his middle name. We also examined whether conservatives primed with concrete religious words would rate President Obama more negatively and be more likely to view him as Muslim than those primed with other word types. Furthermore, we predicted that those who mis-identify President Obama as Muslim would rate him more negatively than would those who view him as Christian. The results provided mixed support for these hypotheses. Conservatives primed with President Obama’s middle name rated him significantly more negatively than did those in the control condition. This effect was not found for politically liberal or moderate participants. Name priming did not significantly affect views of President Obama’s religious affiliation. Although not statistically significant, conservatives primed with abstract religious words tended to rate President Obama more negatively than did those primed with other word types. Religious word priming significantly influenced views of President Obama’s religious affiliation; interestingly, participants primed with abstract religious words were more likely to think President Obama is Muslim than were those primed with religious agent or non-religious words. As predicted, participants who thought president Obama was Muslim rated him significantly more negatively than did those who thought he was Christian. Overall, our results provide some evidence that ethnic name and religious word priming can significantly influence opinions, even with a well-known and specific person.

## Introduction

*“First of all*, *I don’t believe that the guy is a Christian… If you follow his story*, *if you read his book*, *if you understand about Obama-I mean*, *that’s not a Christian name*, *is it*?*”–*Antonio Sabato Jr., July 2016

*“Ultimately though tonight's not about the disagreements Governor Romney and I may have*. *It's what we have in common*, *beginning with our unusual middle names*. *Actually Mitt is his middle name*. *I wish I could use my middle name”–*President Barack Hussein Obama, 67th annual Alfred E. Smith dinner, October 2012

Political discourse in the United States has been described as being marked by incivility [1] with efforts often focused on highlighting a candidate’s similarities with the electorate on one hand, and demonizing political opponents on the other. To that end, candidates and their supporters may use a variety of strategies, such as coded talk [2], to discretely remind voters that they share the same religious beliefs and values as their constituents. For example, conservative evangelical voters are likely to identify a political candidate who uses religious coded talk as an in-group member [2] and to express higher levels of support for that candidate. When Barack Obama entered the 2008 Presidential race he was the first African-American major party candidate and his out-group status, both in terms of his ethnicity and rumored religious (non-Christian) beliefs, was frequently discussed in the media. For example, as illustrated by the quote above, references to President Obama’s middle name were often used in an attempt to sway potential voters. We suspect that the use of religious coded talk and references to the President’s middle name could serve as primes, consequently influencing views of potential voters. The authors are interested in exploring the effects of both ethnic (e.g., name) and religious priming, similar to what may have occurred during the 2008 and 2012 Presidential elections, on people’s attitudes toward President Obama, a well-known figure in contemporary America.

Is there any reason to believe, as suggested by the quotes above, that our beliefs about or attitudes toward a well-known individual may be influenced by something as inconsequential as a name? Prior research has shown that, as social perceivers, humans tend to automatically categorize people based on observable traits and physical features, such as ethnicity, age, and sex [3,4]. However, as demonstrated by Antonio Sabato Jr, an actor who spoke on the opening night of the 2016 Republican National Convention, people may also rely on less obvious cues, such as a person’s name, when engaged in the process of social categorization [5,6].

Previous research has shown that priming participants with names can activate stereotypes [7]. For example, providing foreign or race-typed names can result in prejudicial treatment in the job [8–10] and housing markets [11]. Specifically, job applicants with African American or Arab sounding names were less likely to be contacted for interviews and were evaluated more negatively than were applicants with White sounding names, even when they had equally strong resumes [8–10]. Similarly, Carpusor and Loges [11] found that landlords were less likely to return e-mail inquiries from those with Arab or African American sounding names (e.g. Said Al-Rahman & Tyrell Jackson, respectively) than they were from those with White sounding names (Patrick McDougall).

Throughout his candidacy and Presidency, Barack Obama unwaveringly maintained that he is Christian. However, in spite of his assertions, according to CNN/Opinion Research Corporation interviews [12] of 1012 American adults, when asked to identify President Obama’s religious affiliation, 29% of respondents identified him as being Muslim. Moreover, when the data were analyzed by respondents’ political affiliation differences were found, such that 43% of Republicans, 29% of Independents, and 15% of liberals said he is Muslim [12]. This is consistent with recent Public Policy Polling data [13], which found that 54% of Republican primary voters believed he is Muslim, while only 14% said he is Christian. These findings suggest that factors other than the President’s stated beliefs are influencing perceptions of his religious affiliation. One of these factors may be the use of his middle name, Hussein, which is associated with the Muslim religion, in the media. Because Muslims are viewed significantly more negatively than many other religious and racial minorities in America [14], misidentification of President Obama as Muslim may negatively influence how people view him in other, non-religious, domains.

There is some evidence to suggest that ethnic name priming can negatively influence impressions of President Obama, specifically. In 2010, during President Obama’s first term in office, Waismel-Manor and Stroud [15] had American and Israeli participants watch a video of President Obama discussing Israeli-Palestinian affairs. The video included a brief ticker at the bottom of the screen that identified the president either as “President Barack Obama” or “President Barack Hussein Obama.” Results indicated that Israeli participants who were shown the president’s middle name rated his characteristics and his proposals more negatively than did participants who viewed the video with his middle name excluded. Israeli participants also rated the president as less pro-Israeli when his middle name was included. The middle name manipulation did not significantly influence perceptions of President Obama in the sample of American participants.

Additional research suggests that having pro-diversity beliefs reduces discrimination of individuals with foreign sounding names [16]. Pro-diversity beliefs tend to be related to one’s political ideology. For example, a survey of several European countries showed a negative relationship between right-wing authoritarianism and pro-diversity beliefs [17]. Furthermore, a 2014 poll conducted by the Pew Research Center showed that politically conservative Americans value diversity less than do politically liberal Americans [18]. Presumably, as compared to liberals, politically conservative Americans would have less pro-diversity beliefs, which may contribute to racial discrimination of individuals with foreign sounding names.

According to some researchers [19], religion is being used as a strategic tool to influence voters at higher levels than ever before. Between 2009 and 2010 the amount of media coverage dedicated to religious topics doubled [20]. Furthermore, according to the Pew Research Center [21], six percent of election stories in major news outlets during the 2012 election cycle contained references to religion. For example, in 2012 the conservative leaning Media Research Center published an online article titled “How Network News Has Twisted Obama’s War on Religion into a Conservative War Against Women.” These examples demonstrate that activating people’s religious beliefs is not only a common occurrence in political discourse, but is done with the apparent intent to influence opinions.

Several studies have shown that religious concepts and contexts can influence cognitions and attitudes. Wenger [22] found that when committed Christians were subliminally primed with religious words, they were more likely to apply their religious beliefs when evaluating historical events compared to participants who were not exposed to the primes. Subliminally priming participants with Christian religious words can increase both implicit and explicit negative attitudes towards various out-groups including African Americans, atheists, Muslims, and gay men [23,24] and can increase in-group favoritism [24]. Participants’ religiosity and spirituality did not change the effect of religious priming [23,24]. When participants were assessed as they passed a religious structure, a condition that can be considered a religious situational context prime, they reported more negative views of non-Christians and more conservative political views than those assessed in front of a non-religious structure [19]. Interestingly, this effect was present regardless of participants’ belief in God [25]. Therefore, it appears that simply priming people with religious concepts, iconography, or architecture is enough to increase negative attitudes towards perceived out-groups.

Although studies have demonstrated that priming people with religious words can influence negative attitudes to out-groups, these studies have largely ignored the influence different types of religious words may have [26]. Ritter and Preston [26] found that religious words aren’t conceptually homogeneous. Rather, they found three distinct conceptual categories of religious words (abstract/spiritual, religious agents, & concrete/institutional). Preston and Ritter [27] compared the effects of priming with “religion” (a concrete/institutional prime) and “God” (a religious agent) on prosocial behaviors towards in-group and out-group members in a sample that consisted primarily of white Christian American participants. Interestingly, they found that participants primed with “religion” demonstrated greater prosocial behaviors toward in-group members whereas those primed with “God” showed greater prosocial behaviors toward out-group members. However, in a follow-up study examining an ethnically and religiously diverse sample in Singapore, Ramsay and colleagues [28] found only very limited evidence for differential effects of “religion” and “God” primes on attitudes towards in-group and out-group members. Specifically, they found that female participants exposed to a religion prime demonstrated slightly more negative attitudes towards out-groups than in-groups but this finding was not replicated in a follow-up experiment. The authors cautiously interpreted their gender-specific findings as spurious. Whether the discrepant findings regarding the effects of God and religion primes are the result of differences in dependent variables (behavioral compared to attitudinal), differences in samples, or some other factor is unclear. There is also reason to suspect that abstract and concrete religious primes might have differential effects on attitudes toward out-groups in conservative participants. Luguri, Napier, and Dovidio [29] found that politically conservative participants who were induced to think abstractly demonstrated less explicit prejudice against gays/lesbians, Muslims, and atheists compared to those induced to think concretely. Interestingly, there was no effect of construal level on attitudes in politically liberal participants who had more positive feelings towards out-groups than did conservative participants irrespective of construal condition. Priming with concrete and abstract religious words might differentially activate religious cognitions in conservative individuals in a similar fashion and thereby differentially influence attitudes toward out-groups [26,29]

### Purpose & hypotheses

Several studies have demonstrated that name and religious priming can negatively influence attitudes towards out-groups. However, results regarding the differential effects of priming with different types of religious concepts are unclear. Furthermore, American political news coverage and discourse is often characterized by the use of religious and ethnic messages, which may prime viewers and influence opinions. Specifically, evidence suggests that some political conservatives attempt to influence opinions of President of Obama by referring to his middle name and by priming with religious constructs. However, few empirical studies have been conducted examining how these factors can influence attitudes towards a specific, well-known political figure such as President Obama. Therefore, the purpose of the present study was to examine the influence of religious word priming and racially stereotyped name priming on attitudes towards President Obama. The study examined the following hypotheses:

1. Politically conservative participants primed with President Obama’s middle name would rate him more negatively than would those who are not shown his middle name.
2. Given the discrepant previous results, we did not have a specific directional hypothesis regarding the effect of religious word prime on attitudes toward President Obama. Rather, we sought to investigate whether there would be differential effects of concrete, abstract, religious agent, and non-religious word primes on attitudes toward President Obama in politically conservative participants.
3. Politically conservative participants primed with President Obama’s middle name would be less likely to report that they think the president is Christian than would those who are not shown his middle name
4. We did not have a specific directional hypothesis regarding the effect of religious word priming on opinions of President Obama’s religious affiliation. Rather, we sought to investigate whether there would be differential effects of religious priming word condition on opinions of President Obama’s political affiliation in politically conservative participants.
5. Participants who mis-identify President Obama as Muslim will rate him more negatively as compared to participants who correctly identify him as Christian.

## Method

### Ethics statement

Approval for this study was obtained from the Institutional Review Board (IRB) of the Department of Psychology and Child Development at California State University, Stanislaus (protocol #P-15-048). Participants were treated in accordance with the APA Ethical Principles of Psychologists and Code of Conduct [30]. Consistent with guidelines of the IRB, all participants provided their informed consent electronically by selecting an online checkbox.

### Participants

A total of 336 CSU Stanislaus students (284 females, 51 males, 1 no response) completed this online study. Participants were recruited via the Psychology Department’s online participant pool, and most were compensated with course extra credit. Participants ranged in age from 18 to 62 years (*M* = 22.03 years). One hundred eighty-four participants were Hispanic, 71 were White, 40 were Asian/Pacific Islander, 18 were Black/African American, three were American Indian/Native American, 1 was Arabic, and 18 chose “other” when asked to identify their ethnicity. Four participants were Muslim, 238 were Christian, 2 were Jewish, 2 were Hindu, 7 were Buddhist, 16 were Sikh, 38 were agnostic, non-religious, or atheist, and 28 chose “other” without specifying religious affiliation. When asked about their political identity, 157 participants said they were neutral/moderate, 119 were liberal, and 59 participants considered themselves to be politically conservative.

### Materials

Fifteen religious words (5 abstract, 5 religious agents, & 5 concrete), obtained from Ritter and Preston [26], were utilized in the present study. Additionally, 5 non-religious priming words were utilized in the study for the purposes of creating a control condition (see Table 1 for a complete list of the priming words).

**Table 1:** Religious priming words.

Religious priming word category
**Abstract**
1. Miracle 2. Religion 3. Faith 4. Revelation 5. Belief
**Concrete**
1. Prayer 2. Scripture 3. Sermon 4. Ritual 5. Clergy
**Religious agents**
1. Angel 2. God 3. Soul 4. Spirit 5. Heaven
**Non-religious**
1. Crayon 2. Moon 3. Saturday 4. Book 5. Celebration

Religious priming words taken from Ritter and Preston [26]. For the abstract and concrete religious words, Ritter and Preston used “abstract/spiritual” and “concrete/institutional” labels, respectively. Non-religious words were chosen by the researchers.

Opinions regarding President Obama were examined using an 11-item instrument (see Table 2) based upon questions created by Waismel-Manor and Stroud [15]. This instrument assessed participants’ impressions of Barack Obama’s trustworthiness, competence, honesty, warmth, efficacy, intelligence, fairness, considerateness, peacefulness, and generosity using 6-point Likert scales (greater values indicated higher levels of each construct). Two versions of this instrument were utilized in the study. On one version, President Obama’s middle name was explicitly stated in the instrument instructions as well as in the text of each question. In the other version, his middle name was omitted completely. Because responses to all 10 Likert scale items were highly correlated for both versions (Cronbach’s alphas ≥ .96), responses to all 10 items were averaged to obtain a measure of participants’ overall opinion of President Obama. The instrument included additional categorical item examined participants’ views regarding President Obama’s religious affiliation.

**Table 2:** Opinions of President Obama questionnaire.

Instructions: As you know, Barack Hussein Obama is the current President of the United States. The next few questions are aimed at measuring your impressions and opinions of Barack Hussein Obama. All of the items use a 6-point scale. Please read the items carefully. We appreciate your honest responses.
How would you rate the trustworthiness of President Barack Hussein Obama?
How effective do you think Barack Hussein Obama has been as President?
In your opinion, how competent is President Barack Hussein Obama?
How would you rate the warmth of President Barack Hussein Obama?
How would you rate the honesty of President Barack Hussein Obama?
How would you rate the intelligence of President Barack Hussein Obama?
How would you rate the fairness of President Barack Hussein Obama?
In your opinion, how considerate is President Barack Hussein Obama?
In your opinion, how peaceful is President Barack Hussein Obama?
In your opinion, how generous is President Barack Hussein Obama?
What religious belief do you think President Barack Hussein Obama identifies with? 1. Christian (all denominations) 2. Muslim 3. Jewish 4. Hindu 5. Buddhist 6. Sikh 7. Agnostic/Non-religious/Atheistic 8. Other ____________________

Adapted from questions in Waismel-Manor and Stroud [15]. President Obama’s middle name was omitted in the middle name absent condition.

Religiosity and religious affiliation were assessed using questions adapted from the Brief Multidimensional Measurement of Religiousness/Spirituality [31]. Participant religiosity was assessed using a single question (To what extent do you consider yourself a religious person?) to which participants responded using a Likert-style scale ranging from 1 (not at all) to 7 (very much).

Participant political identity was assessed using a single item taken from Greenwald, Smith, Sriram, Bar-Anan, and Nosek [32]. Participants indicated which one of the seven options provided best described their political ideology. Participants who selected very liberal, moderately liberal, or slightly liberal were categorized as “liberal.” Those who selected slightly conservative, moderately conservative, or very conservative were categorized as “conservative.” Participants categorized as moderate were those who selected “neutral (moderate)” in response to this question. This political ideology question item was included as part of a larger demographic survey that also asked about participants’ age, sex, ethnicity, and other demographic information.

### Design

The study consisted of 2 true IVs (religious word prime and middle name prime). The word prime IV was a between subjects variable with 4 levels (abstract, concrete, religious agents, and non-religious). The prime was accomplished by having participants memorize words from one of the four categories. The name prime was a between subjects variable and consisted of 2 levels (middle name present and middle name absent). Additionally, two participant variables were examined in this study including participant religiosity and political affiliation. This study examined two dependent variables. Overall opinion of President Obama was obtained from the averaged responses to 10 items assessing impressions of the president. Participants’ views regarding President Obama’s religious affiliation were assessed using a single questionnaire item.

### Procedures

This online study was part of a larger study that examined religious priming and explicit attitudes conducted in the spring and fall of 2015 near the end President Obama’s second term in office. Participants were informed that the study would be investigating memory and political attitudes. After giving consent, participants were randomly assigned to one of the four word prime conditions (abstract, concrete, religious agents, and non-religious; see Table 1). In order to direct their attention to the priming words, participants were told that their memory for the words would later be tested. Five words were presented one at a time in random order for five seconds each. Following the presentation of the word primes, participants completed several items assessing explicit attitudes towards various groups (data to be reported elsewhere). Completion of these items took approximately 1–2 minutes. Participants were then shown the same set of priming words presented previously. As with the first presentation of the words, the five words were presented one at a time in random order for five seconds each. Next, participants were randomly assigned to one of the two name prime conditions. Each participant answered a series of questions asking their opinions regarding President Obama. These questions were presented one-at-a time in random order. Religiosity, political affiliation and a variety of demographic information were then assessed. Next, as a part of experimental procedures, recognition for the words presented at the beginning of the study was assessed using a brief word recognition task. Finally, participants were shown the debriefing form.

## Results and discussion

**Fig 1:**
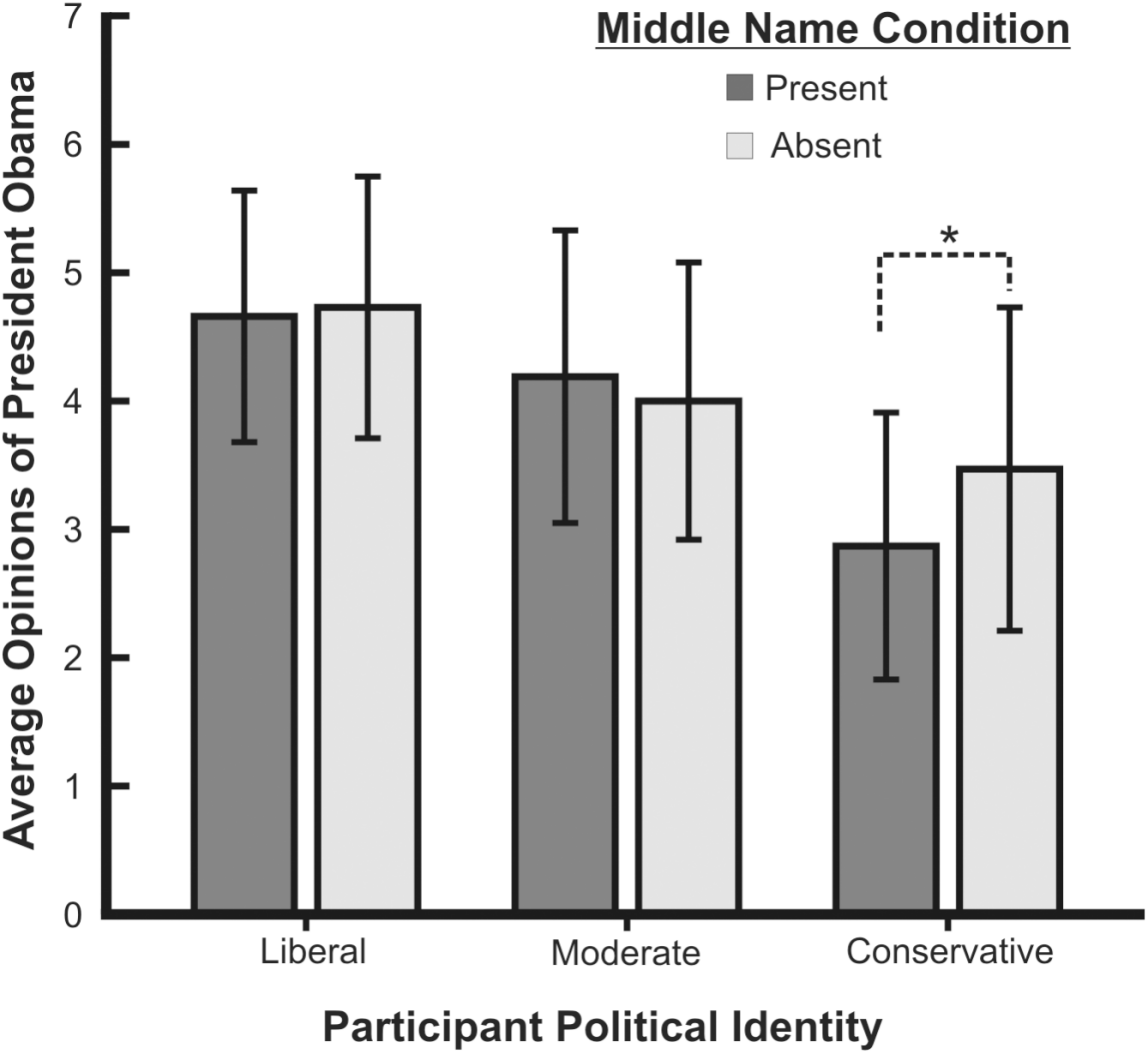
Opinions of President Obama. Average opinions of President Obama for liberal, moderate, and conservative participants. Questions regarding President Obama were asked with the president’s middle name included (gray bars) or absent (white bars). Error bars represent +/- 1 SD. The asterisk (*) indicates the statistically significant pairwise comparison.

First, we conducted a 2 (name prime) x 3 (political identity) ANOVA to test the prediction that conservative participants in the middle name present condition would rate President Obama more negatively than would those in the middle name absent condition. Results showed no significant main effect of the name prime manipulation on opinions of President Obama *F*(1,329) = 1.56, *p* = .213, partial eta-squared = .005. There was, however, a significant main effect of participant political identity on opinions of President Obama, *F*(2, 329) = 39.35, *p* < .001, partial eta-squared = .193. All political identity groups significantly differed, *p*s < .05. Conservative participants (*n* = 59, *M* = 3.12, *SD* = 1.17) rated the president more negatively than did liberal (*n* = 119, *M* = 4.70, *SD* = 1.00) and politically moderate participants (*n* = 157, *M* = 4.09, *SD* = 1.11). The predicted interaction between the name prime manipulation and participant political identity on opinions of the president was marginally significant, *F*(2, 329) = 2.80, *p* = .062, partial eta-squared = .017. Pairwise comparisons showed that conservative participants in the middle name present condition (*n* = 34, *M* = 2.87, *SD* = 1.04) rated President Obama significantly more negatively than did those in the middle name absent condition (*n* = 25, *M* = 3.47, *SD* = 1.26), *p* = .036, *d* = .527 (see Fig 1). Using conventions in psychology this is considered a medium effect size [33]. There was no significant effect of name prime for liberal and moderate participants (*p*s > .28).

The data indicated that, as compared to moderate and liberal participants, conservative participants had more negative views of President Obama. This is not necessarily surprising, as President Obama was presumably not their candidate of choice during the elections and his policies may not be in line with their own. Moreover, the data did provide support for the hypothesis that conservatives’ views of the president would be negatively influenced by the name prime. Politically conservative participants who were shown the president’s middle name tended to rate him more negatively than did those not shown his middle name. The influence of the name prime was not found for politically liberal or moderate participants. These results are consistent with those from previous studies that demonstrated that race-typed names can negatively influence attitudes and behaviors towards minorities and out-groups [7–9,11]. Furthermore, while these data are consistent with previous research specifically demonstrating that President Obama’s middle name can influence people’s views of him, the present study found an effect using an American sample whereas the previous study only found a significant effect in an Israeli sample [15].

One possible explanation for why, in the present study, the effects of the name prime were found only for those participants who were politically conservative is that politically conservative participants may have had low levels of pro-diversity beliefs [17]. By priming those participants with his middle name, it could have served to emphasis the President’s minority status, thereby influencing those participants with lower pro-diversity beliefs to view him more negatively.

To ensure that any differential religious word priming effects were not due to differences in word memorability, a one-way ANOVA was conducted to examine word recognition for the four religious priming word conditions. Results showed that recognition was similar for abstract religious words (*n* = 84, *M* = 4.68, *SD* = 0.82), religious agent words (*n* = 83, *M* = 4.78, *SD* = 0.83), concrete religious words (*n* = 85, *M* = 4.66, *SD* = 0.75), and non-religious words (*n* = 83, *M* = 4.86, *SD* = 0.73), *F*(3, 335) = 1.37, *p* = .251.

Next, we examined the effects of religious word priming condition on politically conservative participants’ opinions of President Obama. To test this, we conducted a 4 (religious word prime) x 3 (political identity) ANCOVA that examined the effects of religious word priming and participant political identity on opinions of President Obama while controlling for participant self-reported religiosity. There was no significant main effect of the religious prime manipulation on opinions of President Obama, *F*(3, 322) = 0.97, *p* = .409, partial eta-squared = .009. Opinions of President Obama were similar for participants who were primed with abstract religious words (*n* = 84, *M* = 4.07, *SD* = 1.20), religious agents (*n* = 83, *M* = 4.16, *SD* = 1.22), concrete religious words (*n* = 85, *M* = 4.08, *SD* = 1.35), and non-religious words (*n* = 83, *M* = 4.24, *SD* = 1.06). The main effect of participant political identity remained significant while controlling for participant religiosity, *F*(2, 322) = 43.98, *p* < .001, partial eta-squared = .215. There was no significant interaction between the religious prime manipulation and participant political identity, *F*(6, 322) = 0.49, *p* = .815, partial eta-squared = .009. Although the differences were not significant, conservative participants primed with abstract religious words (*n* = 11, *M* = 2.88, *SD* = 0.84) tended to rate President Obama more negatively than did those primed with religious agents (*n* = 14, *M* = 3.04, *SD* = 1.25), concrete religious words (*n* = 20, *M* = 3.02, *SD* = 1.26), and non-religious words (*n* = 14, *M* = 3.55, *SD* = 1.19). Therefore, we did not find differential effects of religious word type on attitudes toward President Obama. These findings are inconsistent with those from those of previous studies that demonstrated that priming with religious concepts increases negative attitudes towards out-groups [22–25]. Furthermore, similar to the findings of Ramsay and colleagues [28], our findings are also inconsistent with those that showed differential effects of priming with religion and God concepts [27].

Table 3 shows views of President Obama’s religion as a function of participant political identity, religious word prime condition, and middle name prime condition. We predicted that politically conservative participants primed with President Obama’s middle name would be less likely to report that they think the president is Christian than would those who were not shown his middle name. We also examined the effect of religious word priming condition on opinions of President Obama’s religious affiliation in politically conservative participants. Logistic regression analysis was conducted to test whether or not views of President Obama’s religious affiliation were predicted by name prime, religious word prime, and participant political identity (see Table 4). A test of the full model indicated that the predictors as a set reliably predicted views of President Obama’s religious affiliation, Chi-square (14) = 33.63, *p* = .002. Participant political identity significantly predicted views of President Obama’s religion, Chi-square (2) = 14.91, *p* = .001. Specifically, politically conservative participants were more likely to say that president Obama is Muslim than were politically moderate (Chi-square (1) = 7.23, *p* = .007) and politically liberal (Chi-square (1) = 13.95, *p* < .001) participants. Religious priming word condition also significantly predicted views of President Obama’s religion, Chi-square (3) = 11.11, *p* = .011. Analysis of the data using the concrete word group as the comparison revealed that none of the word priming conditions differed significantly from the concrete word priming condition (all *p*s > .081). When the data were analyzed with the abstract word group as the comparison, results showed that participants primed with abstract religious words were significantly more likely to view President Obama as Muslim than were those shown religious agent words (Chi-square (1) = 6.77, *p* = .009) and non-religious words (Chi-square (1) = 8.10, *p* = .004). Name priming did not significantly predict views of President Obama’s religion (Chi-square (1) = 0.08, *p* = .78) and there were no significant interactions between predictors (all *p*s > .19).

**Table 3:** Summary of participant opinions of President Obama’s religious affiliation.

	Opinion of President Obama’s religious affiliation			
Variable	Christian	Muslim	Other religion	Atheist/Agnostic
**Political identity**				
Liberal	80.7% (96)	11.8% (14)	5.9% (7)	1.7% (2)
Moderate	70.1% (110)	19.1% (30)	6.4% (10)	4.5% (7)
Conservative	58.6% (34)	36.2% (21)	1.7% (1)	3.4% (2)
**Religious Word Prime**				
Concrete	67.1% (57)	21.1% (18)	7.1% (6)	4.7% (4)
Abstract	58.3% (49)	31% (26)	6% (5)	4.8% (4)
Agent	79.5% (66)	14.5% (12)	3.6% (3)	2.4% (2)
Non-religious	81.9% (68)	12% (10)	4.8% (4)	1.2% (1)
**Name prime**				
Name present	70.7% (118)	22.2% (37)	5.4% (9)	1.8% (3)
Name absent	72.6% (122)	17.3% (29)	5.4% (9)	4.8% (8)
**Overall**	71.4% (240)	19.6% (66)	5.4% (18)	3.3% (11)

Values are percentages (frequencies) within each condition. Participants who selected any religious category other than Christian or Muslim were placed in the other religion category.

**Table 4:** Summary of logistic regression analysis for variables predicting opinions of President Obama’s religion.

Variable	Beta (SE)	Odds ratio (95% CI)
**Political identity**		
Moderate	-1.00 (0.37)	0.37 (0.18–0.76)
Liberal	-1.73 (0.46)	0.18 (0.07–0.44)
**Religious priming word**		
Non-religious^a^	-0.92 (0.52)	0.40 (0.14–1.12)
Abstract^a^	0.58 (0.40)	1.79 (0.81–3.94)
Agents^a^	-0.60 (0.45)	0.55 (0.23–1.33)
Non-religious^b^	-1.50 (0.53)	0.22 (0.08–0.63)
Agents^b^	-1.18 (0.45)	0.31 (0.13–0.75)
**Name prime**	-0.09 (0.32)	0.92 (0.49–1.71)

The outcome variable (opinion of President Obama’s religion) was coded as 0 = “Christian” and 1 = “Muslim.” Conservative was the comparison category for the political ID variable. Middle name present was comparison category for the name prime variable. The model correctly classified 79% of cases and Nagelkerke *R*^*2*^ indicated that the model explained 16.2% of the variance in views of President Obama’s religion. The Hosmer and Lemeshow goodness of fit test was not significant (Chi-square (8) = 5.83, *p* = .67) indicating that model fit the data well.

^a^Concrete and

^b^abstract were the comparison categories for the religious priming word variable.

The data revealed that religious word priming did affect views of President Obama’s religious affiliation. Interestingly, participants primed with abstract religious words were less likely to think that the president is Christian than were those primed with concrete religious, religious agents, or non-religious words.

Finally, to test the hypothesis that those who believed that President Obama is Muslim would rate him more negatively than would those who believe he is Christian, an independent samples *t*-test not assuming equal variances was conducted. As predicted, the test showed that those who thought president Obama is Muslim (*n* = 66, *M* = 3.71, *SD* = 1.35) did, in fact, rate him significantly more negatively than did those who thought he was Christian (*n* = 240, *M* = 4.28, *SD* = 1.14), *t*(91.97) = 3.17, *p* = .002, *d* = 0.46. These results are consistent with findings that Muslims are viewed negatively in America [14]. There are a variety of reasons for these anti-Muslim attitudes and behaviors, including fear, misunderstanding, and the fact that many recent terrorist acts have been committed by members of extreme factions identifying themselves as Muslim [34]. Therefore, based on the generally negative attitudes toward Muslims, it is reasonable to expect that those who believe President Obama is Muslim will hold more negative attitudes toward him.

This study had several limitations. Due to our necessary reliance on a convenience sample, we had a relatively small number of politically conservative participants. Furthermore, politically conservative college students might not represent conservatives in the general population. Research has shown that when diversity is actively enacted and promoted at universities with diverse student populations, such as the one at which these data were collected, there are several benefits including students’ personal growth, cultural understanding, and commitment to understanding others [35]. This increased cultural understanding could provide a buffer against the negative impacts of racial and religious primes. Therefore, the politically conservative college students in the present study might not be as influenced by the effects of racial and religious primes as would political conservatives in the general population. Another limitation could have been the manner in which we presented the religious word prime. Our word memory task did not require that participants process the meanings of the priming words. Potentially, a task which requires that students attend to the meaning of the priming words could influence participants to employ either concrete or abstract modes of thinking which could, in turn influence attitudes towards out-groups. Additionally, we did not administer a pro-diversity beliefs instrument and so we were unable to directly ascertain whether or not this variable plays a role in how priming impacts attitudes. Although our sample primarily Christian, the religious priming words utilized in the study could also be viewed as a limitation because they might not be relevant to non-Christian participants. Finally, we did not probe participants for hypothesis awareness. Therefore, it is possible that our results were influenced by demand characteristics.

## Conclusions

Not surprisingly, as compared to politically moderate and liberal participants, politically conservative participants had more negative views of a politically liberal president. Interestingly, conservatives were somewhat susceptible to the negative effects of race-type name priming whereas race-type name priming did not influence views of liberal and moderate participants. Among conservative participants, those who were primed with President Obama’s middle name had more negative views of him, as compared to conservatives not primed with his middle name.

The influence of religious word priming was less straightforward. Although participants primed with non-religious words tended to have more positive views of the president and were more likely to correctly identify him as Christian, priming conservative participants with abstract religious words did not reduce prejudicial views. Rather, similar to Ramsay et al. [28], we did not find significant differences between religious priming word types on opinions of President Obama. However, participants in the abstract word condition, irrespective of political identity, were more likely to mis-identify him as Muslim. Whether these surprising findings resulted from something specific to this study (e.g. the manner in which religious word priming was accomplished in the study) or reflects the actual nature of the influence of religious word priming is unclear and may be investigated in future studies. Given the inconsistent findings regarding the influence of religious priming words, more research in this areas is needed.

Additionally, conservative participants were more likely to think that President Obama is not Christian than were moderate and liberal participants. Specifically, conservative participants were more likely to mis-identify him as Muslim. Finally, those participants who mis-identified the president as Muslim, had less positive views of him, as compared to participants who correctly identified him as Christian. Taken together, these findings support the idea that even though he is a well-known public figure, attitudes toward President Obama can be influenced by subtle things like name and religious primes.

Given the contentious political climate in the United States, future research should continue to investigate factors that influence attitudes toward public figures. By strategically using names or religious concepts and iconography, it may be possible to create the perception of a political opponent being a member of an out-group. Furthermore, we suggest investigating the role of pro-diversity beliefs on attitudes of in-group and out-group members. Therefore, future research may focus on the ways in which in-group/out-group perceptions affect attitudes, likelihood of voting, and perceptions of political legitimacy.

## Supporting information

S1 File Obama name and religious prime data. (XLSX)
